# Supplementary material for: The Role of Cytokinins during the Development of Strawberry Flowers and Receptacles
Source: Plants (Basel). 2023 Oct 25;12(21):3672. doi: 10.3390/plants12213672 (PMC10649685; doi:10.3390/plants12213672)
Supplement: Supplementary file 1 [file plants-12-03672-s001.zip › plants-2639526-supplementary.pdf]

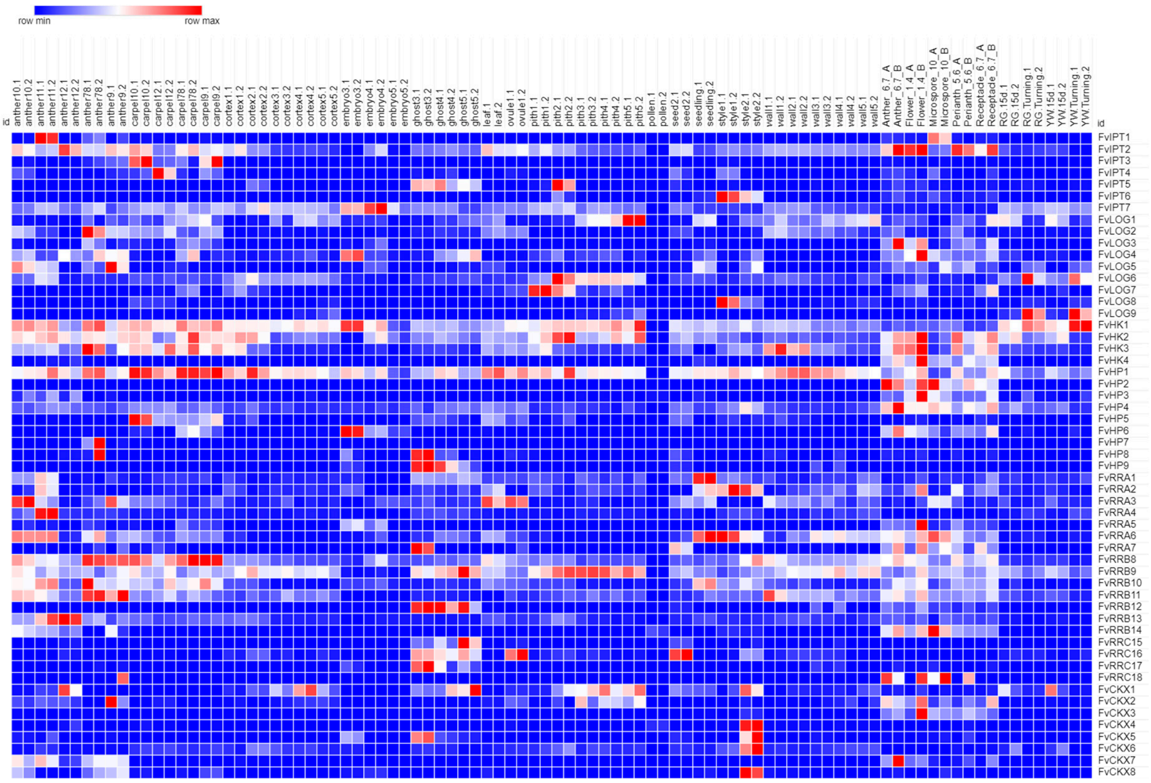

Figure S1. Heat map depicting transcriptome data obtain from different *F. vesca* organs and tissues at different developmental stages. Anthers, Carpel, Cortex, Embryo, Ghost, Leaf, Ovule, Pith, Pollen, Seed, Seedling, Style, Wall, Flower, Microspore, Perianth, and Receptacles(young 15 days after anthesis and turning from Rugen (RG) and Yellow wonder (YW) varieties). Transcriptomic data was obtained from Hawkins et al., 2017 [29].

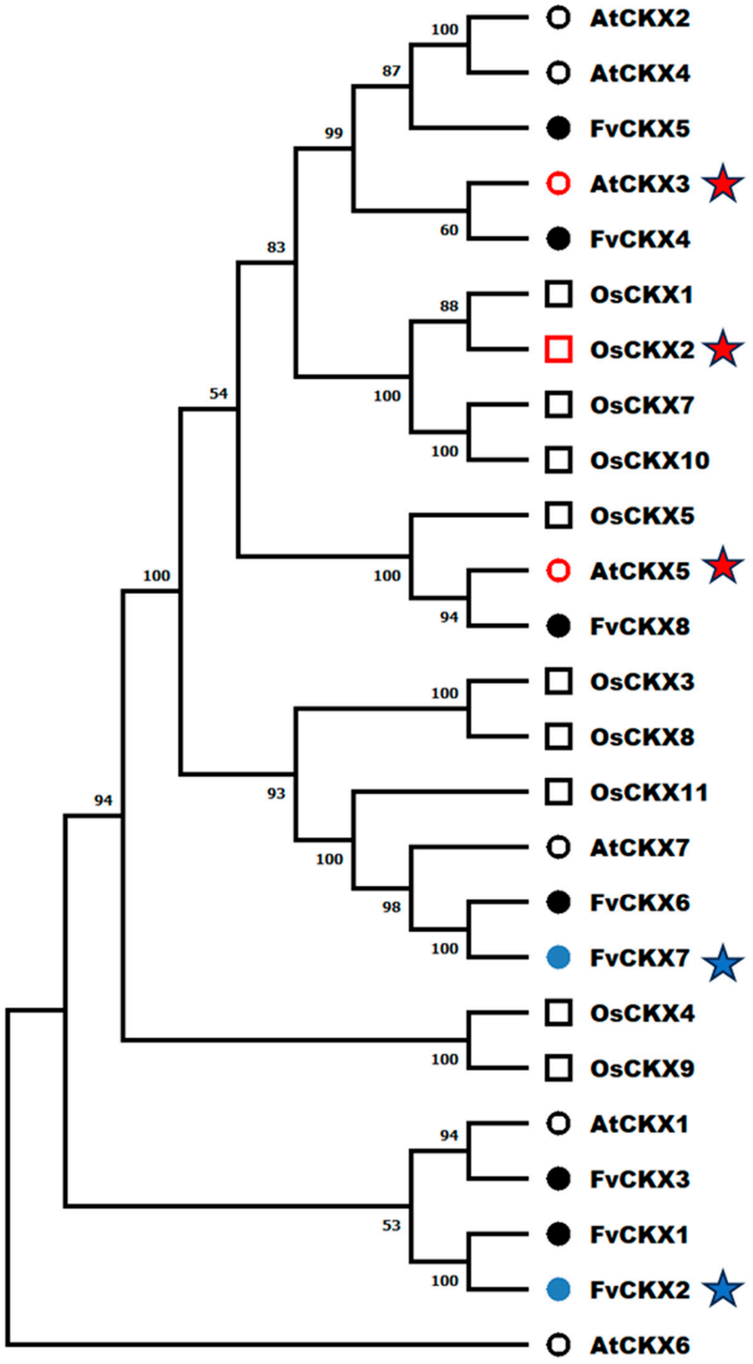

Figure S2. Phylogenetic tree with CKX proteins sequence from *Arabidopsis thaliana* (At), *Oryza sativa* (Os), and *Fragaria vesca* (Fv). Red stars indicate *A. thaliana* and *O. sativa* genes for which loss of function mutations result in larger organs and higher yield. Blue stars indicate the *F. vesca CKXs* homologues to those expressed in *F.* × *ananassa* receptacles as found by ISH. Not all *CKX* genes were assayed.

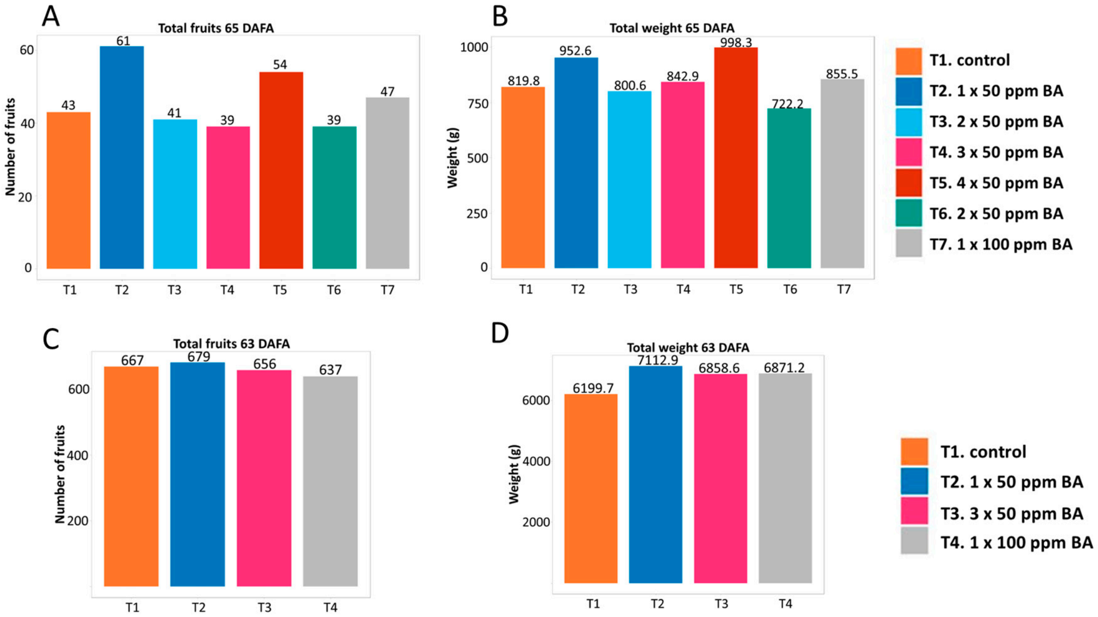

Figure S3. Strawberry yield data. Total number of fruits produced per treatment A) 65 DAFA fall-winter and C) 63 DAFA spring-summer. Total weight of the produced fruits per treatment B) 65 DAFA fall-winter and D) 63 DAFA spring-summer. Days after first application (DAFA).
